# Authors’ reply

**Published:** 2010

**Authors:** Vipul Arora, Kim R Usha, Hadi M Khazei

**Affiliations:** Orbit, Oculoplasty and Oncology Clinic, Aravind Eye Hospital and Postgraduate Institute of Ophthalmology, Madurai, Tamil Nadu, India

Dear Editor,

The authors wish to thank Gupta *et al*.[1] for their interest in our article[2] and for their comments. Point-wise clarification of the queries is given herewith:

1. In this case, we can observe prominent veins and thinning of skin with some hypopigmented lesions, which in itself is suggestive of hypoplasia. Moreover, the texture of the skin was felt to be hypoplastic and it is difficult to notice it in a two-dimensional image.
2. We do not agree with the statement that only microphthalmia is the feature of Delleman syndrome, as the authors[3] themselves and other authors who have studied and compared their cases with previous reported cases have always mentioned anophthalmia / microphthalmia as a feature of the oculocerebrocutaneous syndrome. Hennekam[4] and Moog et al.[5] have also described multiple features associated with this syndrome.Therefore we still believe that this is a case of Delleman Oorthuys syndrome.
3. The third image was only included as per recommendations of a peer reviewer and there was no recurrence of cystic swelling as the case is still on follow-up. However, we do agree that the gradually expanding custom-made prosthesis is recommended in such cases, which is being followed in this case as well.
